# Seroepidemiological Survey on the Impact of Smoking on SARS-CoV-2 Infection and COVID-19 Outcomes: Protocol for the Troina Study

**DOI:** 10.2196/32285

**Published:** 2021-11-22

**Authors:** Riccardo Polosa, Venera Tomaselli, Pietro Ferrara, Alba Corina Romeo, Sonja Rust, Daniela Saitta, Filippo Caraci, Corrado Romano, Murugesan Thangaraju, Pietro Zuccarello, Jed Rose, Giulio Giacomo Cantone, Margherita Ferrante, Jonathan Belsey, Fabio Cibella, Elisa Interlandi, Raffaele Ferri

**Affiliations:** 1 Institute of Internal Medicine Azienda Ospedaliera Universitaria “Policlinico - V. Emanuele” Catania Italy; 2 Department of Clinical & Experimental Medicine University of Catania Catania Italy; 3 Center of Excellence for the Acceleration of Harm Reduction Università di Catania Catania Italy; 4 Department of Political and Social Sciences University of Catania Catania Italy; 5 Center for Public Health Research University of Milano–Bicocca Monza Italy; 6 Value-based Healthcare Unit Research Institute, IRCCS MultiMedica Milan Italy; 7 Oasi Research Institute IRCCS Troina Italy; 8 Department of Drug and Health Sciences University of Catania Catania Italy; 9 Bioanalytical Laboratory Center for Smoking Cessation Duke University Medical Center Durham, NC United States; 10 Department of Psychiatry and Behavioral Sciences Duke University Medical Center Durham, NC United States; 11 Department of Medical Surgical Sciences and Advanced Technologies “G.F. Ingrassia” University of Catania Catania Italy; 12 Department of Physics and Astronomy, Ettore Majorana University of Catania Catania Italy; 13 JB Medical Ltd Sudbury United Kingdom; 14 National Research Council of Italy Institute of Biomedicine and Molecular Immunology Palermo Italy; 15 Federazione Sanità (CIDEC) Sicilia Italy

**Keywords:** antibody persistence, cotinine, COVID-19, SARS-CoV-2, seroprevalence, smoking impact, smoking status

## Abstract

**Background:**

After the global spread of SARS-CoV-2, research has highlighted several aspects of the pandemic, focusing on clinical features and risk factors associated with infection and disease severity. However, emerging results on the role of smoking in SARS-CoV-2 infection susceptibility or COVID-19 outcomes are conflicting, and their robustness remains uncertain.

**Objective:**

In this context, this study aims at quantifying the proportion of SARS-CoV-2 antibody seroprevalence, studying the changes in antibody levels over time, and analyzing the association between the biochemically verified smoking status and SARS-CoV-2 infection.

**Methods:**

The research design involves a 6-month prospective cohort study with a serial sampling of the same individuals. Each participant will be surveyed about their demographics and COVID-19–related information, and blood sampling will be collected upon recruitment and at specified follow-up time points (ie, after 8 and 24 weeks). Blood samples will be screened for the presence of SARS-CoV-2–specific antibodies and serum cotinine, being the latter of the principal metabolite of nicotine, which will be used to assess participants’ smoking status.

**Results:**

The study is ongoing. It aims to find a higher antibody prevalence in individuals at high risk for viral exposure (ie, health care personnel) and to refine current estimates on the association between smoking status and SARS-CoV-2/COVID-19.

**Conclusions:**

The added value of this research is that the current smoking status of the population to be studied will be biochemically verified to avoid the bias associated with self-reported smoking status. As such, the results from this survey may provide an actionable metric to study the role of smoking in SARS-CoV-2 infection and COVID-19 outcomes, and therefore to implement the most appropriate public health measures to control the pandemic. Results may also serve as a reference for future clinical research, and the methodology could be exploited in public health sectors and policies.

**International Registered Report Identifier (IRRID):**

DERR1-10.2196/32285

## Introduction

### Overview

SARS-CoV-2 is the novel coronavirus strain that was first reported as a cluster of viral pneumonia cases of unknown etiology in Wuhan, the capital city of Hubei Province in China, on December 31, 2019. The spreading of SARS-CoV-2 reached pandemic proportion in March 2020, and as of April 2021, more than 141 million cases had been confirmed, and more than 3 million fatalities had occurred worldwide [1].

In late February 2020, the first nonimported cases of COVID-19 were identified in Italy. Since then, SARS-CoV-2 spread rapidly to the community, as reported by national health authorities [2]. On May 23, 2020, at the time of project proposal design, the Italian Ministry of Health reported that, of the 229,327 people who had contracted the virus, 57,752 were still positive, of whom 8695 (15%) were hospitalized with symptoms; 572 (1.0%) were admitted in intensive care units (ICUs); and the remaining 48,485 (84%) were self-isolating at home; 32,735 (14.3%) had died, and 138,840 (60.5%) healed on a total of 2,164,426 tested cases [3]. As of April 2021, 3,870,131 cases and 116,927 deaths have been recorded in the country [4].

Since the beginning of the pandemic, the global scientific community was engaged in intense research efforts to understand all aspects of this public health crisis, from the clinical features of the disease and risk factors for adverse outcome, the patterns of viral spread in the population, the role of asymptomatic or subclinical cases in human-to-human transmission, and the serological response. The clinical features of COVID-19 range from a self-limited flu-like syndrome to progressive lung involvement with respiratory failure and widespread systemic effects [5-7]. Epidemiological surveillance has primarily focused on hospitalized patients with severe disease, and as such, the full spectrum of the disease, including the extent and proportion of mild or asymptomatic infections, is less clear. Evidence suggests that asymptomatic or oligosymptomatic infection is not uncommon [8-11]. A recent review reported that at least one third of SARS-CoV-2 infections are asymptomatic and that almost three quarters of persons who are asymptomatic at the time of a positive polymerase chain reaction (PCR) test result will remain asymptomatic [12]. Remarkably, sequelae of previous viral pneumonia have been reported in chest computed tomography scans of asymptomatic individuals [13], and SARS-CoV-2 transmission from asymptomatic cases to others has been documented [13,14].

In this frame, a seroprevalence study is an ideal attempt for measuring the true infection rates in general or specific populations [11,15,16]. In Italy, a nationwide survey was conducted by the National Institute of Statistics, reporting an IgG seroprevalence of 2.5% [17]. However, there is limited use of seroprevalence studies to retrospectively identify potential predictors of infection susceptibility and disease severity, both in the general population and in specific subgroups (eg, smokers, pediatric populations, or older adults).

Several risk factors for severe COVID-19 have been identified, including cardiovascular disease, diabetes, obesity, and chronic obstructive pulmonary disease [18-21]. Intuitively, one important additional risk factor is expected to be cigarette smoking. Smokers have a higher risk for developing viral and bacterial respiratory infections [22-24], being five times more likely to have influenza and twice more likely to develop pneumonia [25].

However, the role of smoking in SARS-CoV-2 infection susceptibility and COVID-19 outcomes is still unclear. Although there appears to be a higher risk for ICU admission and adverse outcomes [26-28], it has been reported that the prevalence of smoking among hospitalized COVID-19 patients is far lower than would be expected based on population smoking prevalence [29-32]. These findings were initially derived from Chinese case series, and although it is possible that the prevalence of smokers in the Chinese case series may be underrepresented due to inaccurate recording of their smoking status, similar findings have been reported in France [30], Germany [33], Italy [34], and the United States [32,35].

It is not clear how far the underrepresentation of smokers among COVID-19 inpatients reflects problems with poor reporting of the smoking status. Given the challenging circumstances of the pandemic, recall or reporting bias cannot be excluded. The possibility for inaccurate recording, false reporting, or underreporting of the smoking status due to the challenging situations at wards/ICUs with work overloads and operating in a persistent state of emergency should not be underestimated. Improving the quality of clinical and behavioral data mandates the need for an accurate and dedicated recording of the smoking status. Alternatively, population-level data collected outside of hospital settings are required. Another important limitation is that most of the observations were unadjusted for smoking-related comorbidities, which are known to be associated with higher risk for an adverse outcome in patients with COVID-19 [18]. Lack of adjustment for relevant confounders means it is not possible to disentangle the effect of smoking. Addressing all these limitations is important for evaluating clinical risk, developing clear public health messages, and identifying targets for intervention.

### Research Objectives

Surveillance of antibody seropositivity in a population can allow inferences to be made about the cumulative incidence of infection in the population. Additionally, little is currently known about antibody kinetics. Asymptomatic infected persons may clear the virus more quickly than do symptomatic patients, and antibody titers in the former are likely to be lower, if they seroconvert at all, than in infected symptomatic patients [11,36]. Furthermore, understanding the association between smoking and SARS-CoV-2 infection susceptibility or COVID-19 outcomes is generally limited and of poor quality.

To summarize, there is a need for robust population-based evidence on the association of smoking with SARS-CoV-2 infection and COVID-19 outcomes, adjusting for potential confounding variables (eg, sociodemographic characteristics, key worker status, and comorbid health conditions), and a population seroprevalence study could be useful for this goal. The following key research questions will be addressed:

- Does smoking increase susceptibility to SARS-CoV-2 infection?
- Does smoking increase susceptibility to COVID-19 outcomes?
- Does smoking affect the serological response after SARS-CoV-2 infection?

### Research Proposal

We propose a 6-month prospective study using in combination a random population sample (taken from residents of the town of Troina; the town with the highest prevalence of positive SARS-CoV-2 cases in Sicily at the time of drafting the protocol in March-May 2020) and a convenience sample (taken from staff of the Troina’s main health care establishment, reported to have high infectious levels in the same time period) to investigate the prevalence of past infection, as determined by seropositivity (anti–SARS-CoV-2–specific IgG by enzyme-linked immunosorbent assay [ELISA]). The biochemically verified smoking status of the study population (ie, serum cotinine) will be correlated with serological data and COVID-19 outcomes (ie, clinical symptoms and hospitalization).

### Study Aim

Epidemiological exposure data and venous blood (for measurements of anti–SARS-CoV-2–specific IgG and serum cotinine levels) will be systematically collected. Demographic, medical history, and epidemiological exposure data will be recorded from specifically designed questionnaires (for COVID-19 outcomes, relevant comorbidities, and smoking status) and shared rapidly in a format that can be easily aggregated, tabulated, and analyzed across many different regional (or national and international) settings for timely estimates of COVID-19 virus infection and its immunologic response rates according to the smoking status, and to inform public health responses and policy decisions.

## Methods

### Specific Aims

In summary, the main objectives of the study will be to:

- Quantify the proportion of SARS-CoV-2–infected people (by serology assessment of anti–SARS-CoV-2 IgG levels) in a random population sample at baseline
- Quantify the proportion of SARS-CoV-2–infected people (by serology assessment of anti–SARS-CoV-2 IgG levels) in a convenience sample (hospital staff) at baseline
- Quantify the proportion of participants with asymptomatic SARS-CoV-2 infection
- Quantify the proportion of mildly-to-moderately symptomatic (management at home) COVID-19 cases among all participants
- Quantify the proportion of severely symptomatic (management at hospital) COVID-19 cases among all participants
- Quantify the proportion of biochemically verified (by serum cotinine) current, former, and never smokers among all participants (random population sample + convenience sample)
- Quantify the proportion and analyze the association between smoking status (current, former, and never smokers) and:
  SARS-CoV-2 infection (asymptomatic and symptomatic combined)
  Asymptomatic SARS-CoV-2 infection
  Mild-to-moderate COVID-19 (management at home)
  Severe COVID-19 (hospitalization needed)
- Record relevant clinical confounders known to be associated with COVID-19 outcomes
- Compare anti–SARS-CoV-2 IgG levels at baseline among current, former, and never smokers
- Monitor and compare anti–SARS-CoV-2 IgG levels changes from baseline to 2 months and 6 months among current, former, and never smokers.

### Study Design

**Figure 1:**
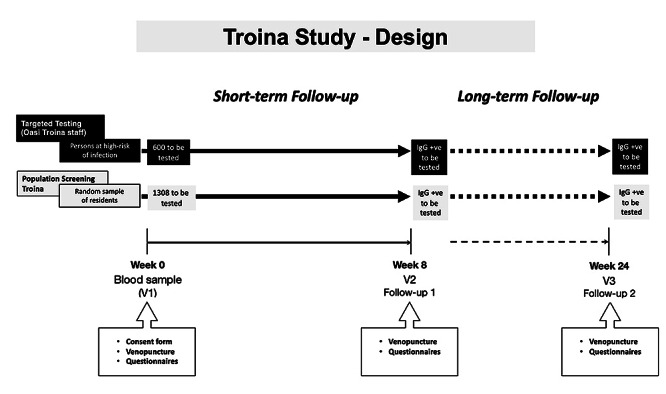
The Troina Study: design.

This study will investigate the association between biochemically verified smoking status of the study population and serological data as well as COVID-19 outcomes (ie, clinical symptoms and hospitalization). The research design involves a 6-month prospective multiple cohort study with serial sampling of the same individuals each time. Sampling will be commenced in July and repeated at 8 weeks (Figure 1). A final follow-up visit will be carried out at 24 weeks.

### Study Population and Setting

Within the geographic scope of the study, high incidence of positive cases was identified in the general population of Troina, a town of around 9000 inhabitants in the province of Enna, in the center of Sicily. This town was hit hardest in terms of COVID-19 cases during the first wave in Italy [2-4], with hundreds of cases registered in the first epidemic weeks in March 2020 [37,38], and was declared a *red zone* on March 29, 2020, with the enforcement of lockdown retractions in that area [38]. The study population will consist of a population-based, age-stratified cohort in Troina that will be sampled through random selection of town residents. Identification and recruitment of participants will expand over different age groups to determine and compare age-specific attack rates. For logistic reasons, specimen and data collection will be performed at a single location, asking participants to travel to that location to participate in the study. Targeted testing will also be extended to a convenience sample consisting of about 600 staff members of Troina’s main health care establishment (high-risk individuals).

### Eligibility Criteria

Any individual identified for recruitment, irrespective of age, can participate. Exclusion criteria will be refusal to provide informed consent or contraindication to venipuncture. Suspected or confirmed active/acute or prior SARS-CoV-2 infection should not be considered as an exclusion criterion for this investigation. Doing so would underestimate the extent of infection in the population. For individuals currently receiving medical care for COVID-19 infection, a family member or proxy may be used to complete the questionnaire on their behalf.

### Smoking Status Definition

Current smokers will be defined as those who report that they smoke and have serum cotinine levels ≥20 ng/mL. Former smokers will be defined as those who report that they used to smoke in the past but not now and have serum cotinine levels <20 ng/mL. Never smokers will be defined as those who report that they never smoked in the past and have serum cotinine levels <20 ng/mL.

### Data Collection

Each participant recruited into the study will be asked to complete a questionnaire that will record the following information: demographics, information about known COVID-19 and relevant clinical course, comorbidities, and smoking status.

### Specimen Collection

A small amount of blood (10 mL) will be collected from each participant upon recruitment (T0) and at specified follow-up time points (T8wks, T24wks).

### Specimen Transport and Biobanking

For each biological sample collected, the time of collection, the conditions for transportation, and the time of arrival at the study laboratory will be recorded. Specimens should reach the laboratory as soon as possible after collection. Serum should be separated from whole blood and stored at –20 °C or lower and shipped on dry ice. A biobanking facility will be established in Troina.

### Sample Storage

Prior to testing, serum samples will be stored at –80 °C at the reference biobanking facility. It is recommended to aliquot samples prior to freezing to minimize freeze thaw cycles.

### Serological Testing

Serologic assays of high sensitivity and specificity for SARS-CoV-2 have been recently validated and published. Serum samples will be screened for the presence of SARS-CoV-2–specific antibodies using a quantitative ELISA test for anti–SARS-CoV-2 IgG (Euroimmun, CND W0105040619) [39].

Serum samples will be stored at –80 °C until use, and the assay will be performed according to the manufacturer’s protocol. The neutralization capability, specificity, and sensitivity of the test have been thoroughly investigated and published together with the assay validation [40]. Reagent wells of the assays are coated with recombinant structural protein (S1 domain) of SARS-CoV-2. The optical density (OD) will be detected at 450 nm, and a ratio of the reading of each sample to the reading of the calibrator, included in the kit, will be calculated for each sample (OD ratio). The cutoff value for IgG OD ratio is 0.3. Following blocking, diluted serum (1:100 or 2-fold serially diluted for titers) will be added and incubated at 37 °C for 1 hour in the 96-well microtiter ELISA plates. Antigen-specific antibodies will be detected using peroxidase-labeled rabbit antihuman IgG and TMB as a substrate. The absorbance of each sample will be measured at 450 nm. Laboratory procedures involving sample manipulation must be carried out in a biosafety cabinet.

### Cotinine Assay

About 1mL of serum will be pipetted into 10 ml tubes and 100 ng/mL of Ortho-cotinine used as internal standard will be added. About 50 µL of 0.1 M aqueous sodium hydroxide solution will then be added to the culture tube followed by 325 µL of chloroform. The tube will be secured with cap and vortex mixed for ~3 minutes (using VX 2500 Multi Tube Vortex Mixer) and centrifuged for ~4 minutes (in Beckmann Allegra centrifuge) at 2500 rpm. Using a glass Pasteur Pipette, the top aqueous layer will be removed and discarded into the hazardous waste container and the organic layer will remain in the tube. About ~100 mg (0.1 g) of anhydrous sodium sulfate will be added to the organic layer and allowed to rest for ~3 minutes (which will allow the sodium sulfate to absorb any water that may be present in the organic layer). In the end, the clear organic layer (with no water) will be carefully removed (without disturbing the settled sodium sulfate), concentrated to ~100 µL vial insert and placed in a gas chromatography (GC) vial. The concentrated sample will be capped and arranged on to an auto sampler tray for GC injection. One microliter (µL) of each sample will be injected into HP-5 Capillary GC Column (0.32 mm ID, 25 m length, and 0.52 µm film thickness; bonded 5% phenyl; and 95% dimethylpolysiloxane) of GC–nitrogen phosphorous detector. The inlet temperature will be 250 ˚C at split-less mode. The initial oven temperature will be 70 ˚C with a 1-minute hold and then increased to 230 ˚C at the rate of 25 ˚C per minute. Every batch of samples will be run with 6 calibration levels (20, 50, 100, 200, 400, 600 ng/mL), 4 quality controls (20, 100, 400, 600 ng/mL), and 1 blank control for accurate quantification. The amount of cotinine will be reported in ng/mL. The limit of quantification of cotinine is 20 ng/mL.

### Statistical Plan

Estimates of margin of error as a function of seroprevalence are low for 300 samples. We will be aiming for >1000 samples of a representative sample of the population by gender and age groups (0-17 years, 18-65 years, and 66 years and older).

For the enrollment of participants into the study, the following inclusion and exclusion criteria need to be fulfilled:

- Inclusion in population criteria:
  Aged into the aforementioned groups
  Living in the town of interest
  Willing to participate in the baseline and to be recontacted for follow-up waves
- Exclusion from population criteria:
  Persons belonging to the institutionalized population
- Exclusion from sample (censoring) criteria:
  Moved to another village during the study period
  Those stating explicitly their wish not to be recontacted for this study

To correctly represent the population involved, the age groups of interest have been assigned to three different categories by gender in terms of population size. A corresponding targeted sample size for this study is specified for each category.

In the entire population, we estimated several sample sizes depending on the margin of error equal to 0.03, 0.04, or 0.05 for estimate proportions of the sampled population, as shown in Table 1.

**Table 1:** The Troina Study: sample size calculation.

Age groups and gender			Participants, n		Margin of error 3%^a^		Margin of error 4%^b^		Margin of error 5%^c^
**0-17 years**									
	Male	694		100		47		29	
	Female	648		93		44		27	
	Total	1342		193		90		57	
**18-65 years**									
	Male	2751		396		185		116	
	Female	2803		403		189		118	
	Total	5554		799		374		235	
**>65 years**									
	Male	961		138		65		41	
	Female	1237		178		83		52	
	Total	2198		316		148		93	
**Total**									
	Male	4406		634		297		186	
	Female	4688		674		316		198	
	Total	9094		1308		613		384	

^a^(N * 1308) / total (N=9094) = n.

^b^(N * 613) / total (N=9094) = n.

^c^(N * 384) / total (N=9094) = n.

We will draw a multilayered sample with a confidence level of 0.97% to minimize the margin of error to 3% and ensure the best reliability of the sample data. The planned total sample size comprises up to 1308 participants at the recruitment stage. The attrition rate is estimated at 10%.

Information will be collected in a standardized format according to the questionnaires and tools in the protocol. The data shared should include only the study identification number and not any personal identifiable information. We will report the following information:

- The number of households and the number of individuals included
- The age and sex of all individuals included
- The antibody levels in the sample of all individuals included
- The number of individuals with serologic evidence of COVID-19 virus infection (stratified by age)
- The number of individuals with serologic evidence of COVID-19 virus infection who have reported symptoms
- The number of individuals with known previous SARS-CoV-2 infection who were hospitalized or recovered at home
- The number of individuals with serologic evidence of COVID-19 virus infection who are current, former, and never smokers (biochemically verified by serum cotinine assay)

Sociodemographic and baseline characteristics will be summarized for the Troina population, for the convenience sample, and for the total sample recruited. Categorical variables will be reported as numbers and proportions with 95% CIs. Between-group comparisons will be carried out using chi-square testing or Fisher exact test, as appropriate. Continuous variables will be reported as means and SDs, and as medians and IQRs. Between-group comparisons will be carried out using tests like analysis of variance, Mann-Whitney *U* test, or Wilcoxon signed rank test, as appropriate. The proportions of patients developing a positive PCR test for SARS-CoV-2 result over the course of the study will be reported as numbers and proportions with 95% CIs, separated by subgroups identifying those with symptomatic or asymptomatic infection. Data on change in antibody levels from baseline to follow-up will be presented for the whole recruited population. Statistical significance of change will be estimated using repeated-measures *t* testing. Correlation between smoking status and the risk of COVID-19 infection will be tested using a 3 × 2 chi-square test of independence. A *P* value ≤.05 will be considered to represent the threshold of statistical significance for all comparisons.

### Data Collection Procedures

To accomplish the specific aims of the project, data will be stored in a database system after each completed collection step and transferred to central data management for further data processing and merging. Each participant will be assigned a unique identification code consisting of a number identifier that will be used for data merging. After data collection, data validation checks will be performed as agreed in a data validation plan, and data cleaning procedures will be used as applicable to ensure the best achievable quality of data for analysis purposes. Only the study staff working with the fieldwork provider will be able to identify the participants based on the identification codes. Only deidentified data will be transferred from the fieldwork provider to the central data management. All electronic data files will be kept on secure servers with backup processes in place. Personal data of participants must be strictly kept separately from study data and is accessed only by authorized staff for the purposes of the study conduct. Before starting data collection, the involved staff will receive training on the background and objectives of this study, on eligibility criteria, on the participant selection procedure, on ethical obligations, on completion and validation of the procedure, and on the data collection platform. Local fieldwork staff will be trained for each relevant data collection process and logistic-related procedure.

Eligible participants are informed about the study purpose, their requested tasks, time of involvement, data confidentiality, and data protection. Once they stated their willingness to participate, they will proceed with the screening and study enrollment. Enrolled participants have the right to stop their participation at any time without any penalty. Preferably, the reason for the premature end of participation will be recorded. Participants who drop out will not be replaced. Every effort will be made to protect participant confidentiality according to the General Data Protection Regulation.

## Results

We expect to find a higher prevalence of antibodies in individuals at high risk for viral exposure (ie, health care personnel and other essential workers) according to previous evidence and to refine current estimates on the association between smoking status and SARS-CoV-2/COVID-19. A total of 1785 participants have been enrolled in the study. Data cleaning and analyses are ongoing.

## Discussion

This project is the first population-based study that uses seroprevalence data and an objective assessment of the current smoking status to examine the association between smoking and SARS-CoV-2 infection susceptibility and severity. Additionally, the study will examine for the first time the magnitude of the seroconversion response in current smokers, compared to former and never smokers, and the changes in antibody titers over time according to the smoking status. Instead of focusing on hospitalized patients only, the study will also include participants who infected individuals who were either asymptomatic or had COVID-19 but were not hospitalized. It will also consider confounding factors in the association between smoking and SARS-CoV-2 that were not addressed in previous research. Finally, the results from this survey may serve as a reference to other contexts and provide an actionable metric to quantify and offer a clear overview of SARS-CoV-2 spread, and the methodology and findings can be exploited in public health research and policies to minimize the disease impact and implement the most appropriate prevention measures to protect susceptible populations.

## Abbreviations

ELISA: enzyme-linked immunosorbent assay
GC: gas chromatography
ICU: intensive care unit
OD: optical density
PCR: polymerase chain reaction
